# Parsimonious estimation of signal detection models from confidence ratings

**DOI:** 10.3758/s13428-019-01231-3

**Published:** 2019-05-08

**Authors:** Ravi Selker, Don van den Bergh, Amy H. Criss, Eric-Jan Wagenmakers

**Affiliations:** 1 Department of Psychological Methods, University of Amsterdam, Postbus 15906, 1001 NK Amsterdam, The Netherlands; 2 Syracuse University, Syracuse, NY USA

**Keywords:** Signal detection theory, Confidence ratings, Bayesian hierarchical models

## Abstract

Signal detection theory (SDT) is used to quantify people’s ability and bias in discriminating stimuli. The ability to detect a stimulus is often measured through confidence ratings. In SDT models, the use of confidence ratings necessitates the estimation of confidence category thresholds, a requirement that can easily result in models that are overly complex. As a parsimonious alternative, we propose a threshold SDT model that estimates these category thresholds using only two parameters. We fit the model to data from Pratte et al. (*Journal of Experimental Psychology: Learning, Memory, and Cognition, 36*, 224–232 2010) and illustrate its benefits over previous threshold SDT models.

Our ability to recognize stimuli allows us to interact smoothly with the world. We know that if we want to drink water it is a good idea to poor it into a cup instead of onto a piece of paper. We also know that if we want to write something down it is a good idea to use a pen instead of a yoga mat. Although recognizing stimuli is sometimes straightforward, often it is not. Most of the times, our ability to recognize a stimulus is accompanied by a certain amount of noise. When picking mushrooms, it can be hard to distinguish between the mushrooms you can use to top your beautiful saffron risotto, and the mushrooms that will turn your dinner party into the next Jonestown. Not only do eatable and poisonous mushrooms differ in perceptual similarity—it is easy to classify a mushroom with a red cap and white spots as poisonous, but difficult to do so for a poisonous mushroom that looks similar to a common white button mushroom—but the amount of risk involved in making the wrong decision can also differ between situations: when you are starving you might decide to eat a suspicious looking mushroom sooner than when you just had a full course meal. Signal detection theory (SDT; Tanner & Swets, 1954; Green & Swets, 1966) disentangles these aspects of recognition by providing different parameters: (1) the amount of information that is available in the stimulus, and (2) the threshold you set for making one or the other decision.

**Table 1:**
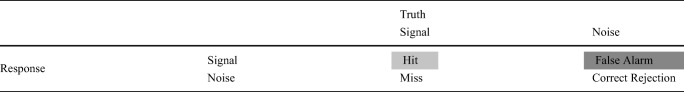
Possible outcomes when trying to discriminate signal from noise stimuli

The rows represent the estimates and the columns represent the truth

In order to separately estimate these two aspects of recognition, an SDT model needs two pieces of information: (1) the proportion of correctly identified signal stimuli (hit rate, HR; the proportion of poisonous mushrooms that were correctly identified as poisonous), and (2) the proportion of incorrectly identified noise stimuli (false alarm rate, FAR; the proportion of non-poisonous mushrooms that were incorrectly identified as poisonous). Table 1 depicts the four possible outcomes when discriminating two types of stimuli; Eqs. 1 and 2 show how these outcomes can be converted to hit rate and false alarm rate:

1 $$ \begin{array}{@{}rcl@{}} \text{Hit Rate} &=& \frac{\text{Hits}}{\text{Hits} + \text{Misses}},  \end{array} $$

2 $$ \begin{array}{@{}rcl@{}} \text{False Alarm Rate} &=& \frac{\text{False Alarms}}{\text{False Alarms} + \text{Correct Rejections}} . \end{array} $$

SDT is a popular model for the analysis of experiments in recognition memory. The most common experiment in this field first requires that participants study a list of words (i.e., the study list). Following a retention interval, participants are presented with another (i.e., the test list, containing words from the study list and new words). For each word on the test list, participants are asked to decide whether the word was from the study list (i.e., ‘old’), or not (i.e., ‘new’). Figure 1 illustrates how the SDT model uses hit and false alarm rates to identify the strength of the signal, *d*^′^, in this task and the threshold, *λ*, that is set to make one or the other decision. To estimate these two parameters, the model assumes that both the signal (i.e., ‘old’ words) and the noise (i.e., ‘new’ words) stimuli can be placed on a latent continuous scale of familiarity. The latent scores are drawn from a signal normal distribution or a noise normal distribution and *d*^′^ represents the difference in means of these distributions. To translate the latent familiarity scores into the dichotomous decision, the model assumes there is a threshold, *λ*, and if the familiarity is lower than that threshold people classify the stimulus as noise while if the familiarity is higher than the threshold people will classify the stimulus as signal.

**Fig. 1:**
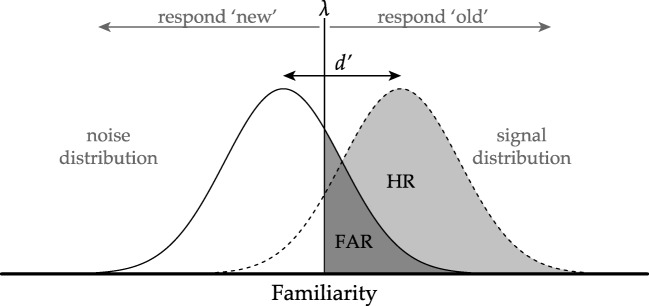
The interaction between the two parameters *d*^′^ and *λ* lead to a certain hit rate (HR) and false alarm rat (FAR). Increasing the decision criterion leads to a lower FAR but also a lower HR, while *d*^′^ stays the same

When estimating only these two parameters, the SDT model has been quite popular (a Google Scholar search for papers published in the last 10 years with keywords ‘signal detection theory’ and ‘psychology’ yielded more than 20,000 results). However, this SDT model assumes that the two distributions have equal variances. Analyses of empirical data in the field of recognition memory, however, often show that the variance of the signal distribution is larger than the variance of the noise distribution (e.g., Macmillan & Creelman, 2005; Swets, 1986; DeCarlo, 2010; Starns & Ratcliff, 2014; Mickes et al.,, 2007). Unfortunately, adding a third parameter *σ* (for the ratio of the variance of the signal-to-noise distribution) to the SDT model creates an identifiability problem; three parameters (i.e., *d*^′^, *λ*, and *σ*) are estimated using only two data points (i.e., hit rate and false alarm rate). To estimate the extra parameter, the model needs more informative data. One way of obtaining more informative data is by having participants rate the familiarity of each item on a confidence rating scale (e.g., “how confident are you that the word presented was on the study list?”, indicated on a Likert scale from 1–7) instead of asking for dichotomous answers (“was the word on the study list or not?”). However, with confidence rating data the number of thresholds that need to be estimated increases with the number of categories. For instance, if the SDT model is fit to data from a four-point Likert scale, this requires estimation of five parameters—*d*^′^, *σ*, and three thresholds—but if the model were fit to data from a ten-point Likert scale, this requires estimation of eleven parameters—*d*^′^, *σ*, and nine thresholds. The estimation of additional thresholds requires larger data sets; to estimate thresholds reliably, it is important that there are a certain number of observations for each category. This in turn means that models with more categories (and therefore more thresholds that need to be estimated) require a larger number of total observations. In recognition memory, accuracy decreases with successive test trials (Criss et al., 2011), limiting the number of observations any individual participant can contribute. This problem is compounded in a typical study where multiple conditions, each requiring many observations, are under investigation simultaneously. Here we introduce a parsimonious method of estimating the thresholds by restricting the way the thresholds can be placed. This parsimony is obtained by modeling thresholds as a linear transformation of ”unbiased” thresholds, which only requires two parameters for any number of thresholds. We estimate parameters in a Bayesian way, and introduce a hierarchical extension to our model that allows the estimation of group-level parameters.

The outline of this paper is as follows. First, we will briefly elaborate on Bayesian methods of parameter estimation. Next, we will introduce our model and the associated receiver operating characteristics (ROC) curves. We will also show how our model leads to Bayesian estimates of detection measures while taking into account the uncertainty of the estimate. Lastly, we will introduce the hierarchical extension and apply the model to memory recognition data from Pratte et al., (2010).

## Modeling the thresholds

**Fig. 2:**
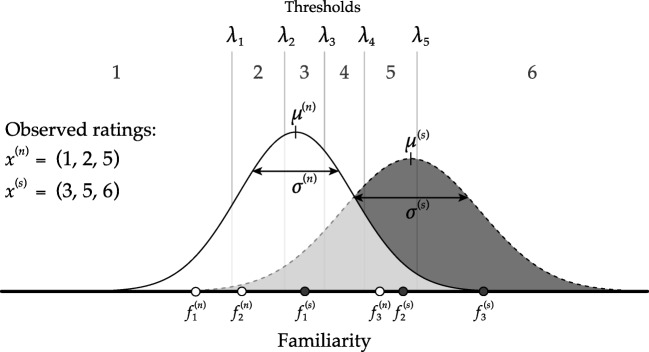
A graphical representation of the SDT threshold model for confidence ratings. Familiarity ratings are drawn from both the noise *f*^(*n*)^ and the signal *f*^(*s*)^ distribution. The associated confidence ratings *x*^(*n*)^ and *x*^(*s*)^ are generated through the thresholds *λ*_*c*_

The key concepts in our SDT threshold model are summarized in Fig. 2. This figure represents an example where an individual observer rated how familiar six items—three signal items and three noise items—are on a Likert scale from one to six. The model describes the process with which these data are generated. The model assumes that the observer makes internal appraisals of the familiarity of the noise items *f*^(*n*)^ and the signal items *f*^(*s*)^, both of which are latent and continuous. These appraisals come from the noise distribution for noise items—a normal distribution with mean *μ*^(*n*)^ and standard deviation *σ*^(*n*)^—or from the signal distribution for signal items—a normal distribution with mean *μ*^(*s*)^ and standard deviation *σ*^(*s*)^. For reasons of identifiability, we assume that the noise distribution is a standard normal distribution; i.e., *μ*^(*n*)^ = 0 and *σ*^(*n*)^ = 1. Equation 3 describes the formal process of this step in the model.

3 $$ f \sim \left\{ \begin{array}{l} \mathcal{N}(0,1)\\ \mathcal{N}(\mu^{(s)},\sigma^{(s)}) \end{array} \quad \text{if} \begin{array}{l} \quad \text{noise \((f^{(n)})\),}\\ \quad \text{signal \((f^{(s)})\).} \end{array}\right.  $$

Once observers have made an internal appraisal of the familiarity of an item, they have to translate this appraisal to the ordinal Likert scale, in this case a scale from one to six. An observer is assumed to accomplish this mapping by placing thresholds *λ*_*c*_ (the *c* represents the order of the threshold) on the latent continuous scale and comparing the internal appraisal with the thresholds resulting in the ratings *x*^(*n*)^ for the noise items and *x*^(*s*)^ for the signal items. As shown in Fig. 2 the internal appraisal of the familiarity of the noise items—$f_{1}^{(n)}$, $f_{2}^{(n)}$, and $f_{3}^{(n)}$—leads to observed ratings *x*^(*n*)^ = (1,2,5), and the internal appraisal of the familiarity of the signal items—$f_{1}^{(s)}$, $f_{2}^{(s)}$, and $f_{3}^{(s)}$—leads to observed ratings *x*^(*s*)^ = (3,5,6).

An important property of the ordinal scale is that the differences between consecutive numbers cannot be assumed equal; on a Likert scale the distance between ‘completely agree’ and ‘agree’ can be larger than the difference between ’agree’ and ’neither agree nor disagree’. Therefore, the translation between the latent continuous appraisal to the ordinal score is relatively lax, and observers are free to use the ordinal scale in different ways. For instance, some observers prefer to use the outer values of the scale while others prefer to use the inner values. To adjust for these individual differences, a proper model needs to be able to estimate the thresholds that are set by an observer to choose a certain answer. In previous SDT models, the number of parameters that needed to be estimated was directly related to the coarseness of the confidence scale that was used (e.g., Morey et al.,, 2008). Consequently, these models are not parsimonious and increase in complexity as the Likert scale becomes less coarse. In addition, the previous approaches are not easily adjusted to incorporate effect of other functional parameters (e.g., a covariate). To arrive at a more efficient way of estimating the thresholds, our model is based on a method introduced by Anders and Batchelder (2013) that uses the Linear in Log Odds function. The Linear in Log Odds function requires only two parameters to estimate a potentially large number of thresholds instead of needing a parameter per threshold (Fox & Tversky, 1995; Gonzalez & Wu, 1999). To estimate *C* thresholds, we first assume a best-guess placement of the thresholds. First, we do so on for the interval [0,1] because it is straightforward to place thresholds in an uninformative way (e.g., the intervals are of equal length). However, since the uncertainty in the SDT threshold model is expressed on the interval [−*∞*,*∞*] we next translate the threshold placement from the [0,1] interval to the [−*∞*,*∞*] interval.^1^

Equation 4 shows how this translation is achieved if we were to assume that *μ*^*s*^ = 1 and *σ*^*s*^ = 1. Equation 5 shows how these ‘unbiased’ thresholds are subsequently translated into the individual ‘biased’ thresholds using a linear transformation.

4 $$ \gamma_{c} = \log{\left( \frac{c/C}{1-c/C}\right)}.  $$

5 $$ {\lambda_{c} = a\gamma_{c} + b.}  $$

**Fig. 3:**
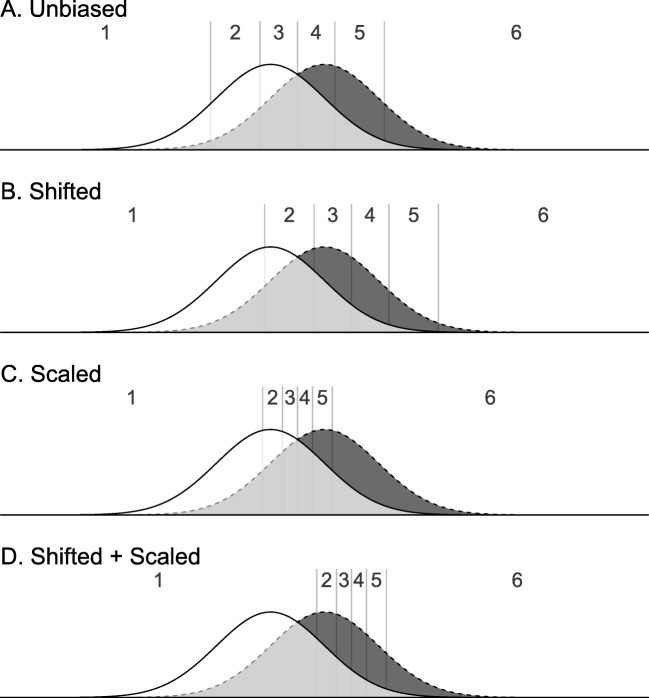
Panel **a** shows the position of the thresholds when an observer is ‘unbiased’, panel **b** shows the position of the thresholds when an observer prefers the lower part of the scale, panel **c** shows the position of the thresholds when an observer is ‘unbiased’ but distinguishes more between values around the center of the scale, and panel **d** shows the position of the thresholds when an observer prefers the lower part and distinguishes more between values where the signal distribution is high and noise distributions is low

Here, *γ*_*c*_ is the unbiased threshold for each position *c* (e.g., *γ*_1_ represents the first unbiased threshold). Scale parameter *a* allows the thresholds to be distributed more closely to the center of the scale or further away from the center of the scale. Shift parameter *b* allows the thresholds to focus more on the left or right side of the scale and could, for example, model response bias. Figure 3 illustrates how these two parameters can result in different threshold placements. Compared to the unbiased thresholds in panel a, panel b shows that the thresholds have shifted to the right, and compared to the thresholds in panel b, panel c shows that the thresholds are placed closer to each other. Compared to panel c, the thresholds in panel d have shifted more to the right. This shows that two parameters can account for many different ways of threshold placement and can be extended to any number of thresholds without requiring additional parameters.

Note that the outer thresholds are always farther away from their neighboring thresholds than the inner thresholds. At first sight this may look like a major assumption of the model, but it is not. The probability of observing a certain rating is not related to the distance between thresholds, but rather to the area under the curve (i.e., the integral from one threshold to the next over either the noise or the signal distribution).

## Bayesian parameter estimation

SDT models have been applied using both classical (Macmillan & Creelman, 2005) and Bayesian frameworks (Rouder & Lu, 2005). In this paper, we adopt the Bayesian framework (Etz et al., 2016; Lee & Wagenmakers, 2013). An important goal of Bayesian statistics is to determine the posterior distribution of the parameters. This distribution expresses the uncertainty of the parameter estimates after observing the data; the more peaked this distribution the more certain the estimate. To obtain the posterior distribution of a parameter (e.g., *d*^′^ or *λ*), the likelihood is multiplied with the prior distribution, see Eq. 6.

6 $$ \underbrace{p(\theta \mid \text{data}, \mathcal{M})}_{{\text{posterior} \atop \text{distribution}}} \overbrace{\propto}^{\text{{proportional to}}} \underbrace{p(\theta \mid \mathcal{M})}_{{\text{prior} \atop \text{distribution}}} \times \ \underbrace{p(\text{data}\mid \theta, \mathcal{M})}_{\text{likelihood}}. $$

In our case, it is not possible to derive the posterior distribution analytically and hence we used MCMC sampling techniques (i.e., implemented in JAGS; Plummer, 2003) to draw samples from the posterior distribution; with enough samples the approximation to the posterior distribution becomes arbitrarily close. As priors, we used normal distributions for all unbounded parameters (mean and shift). For bounded parameters (variances and scale), we used either a gamma prior or a normal distribution truncated from 0 to *∞*. Formal model definitions and prior distributions can be found in the Appendix.

To confirm the performance of the model, we conducted a parameter recovery study. First, we randomly generated 100 values for *μ*^(*s*)^, *σ*^(*s*)^, *a*, and *b*^2^. Each combination of parameters was used to generate ordinal six-point Likert scale data (240 noise and 240 signal items), after which the SDT threshold model was fit to the data. Subsequently, we compared the parameter values used to generate the data with the means of the posterior distributions of the parameter estimates. The correlations between the data generating parameter values and the recovered parameter estimates were high ($r_{\mu ^{(s)}} = 0.96$, $r_{\sigma ^{(s)}} = 0.89$, *r*_*a*_ = 0.99, *r*_*b*_ = 0.98) showing that the SDT threshold model has good parameter recovery. More details on this parameter recovery study can be found in the Supplemental Materials at https://osf.io/ypcqn/.

## ROC curve

**Fig. 4:**
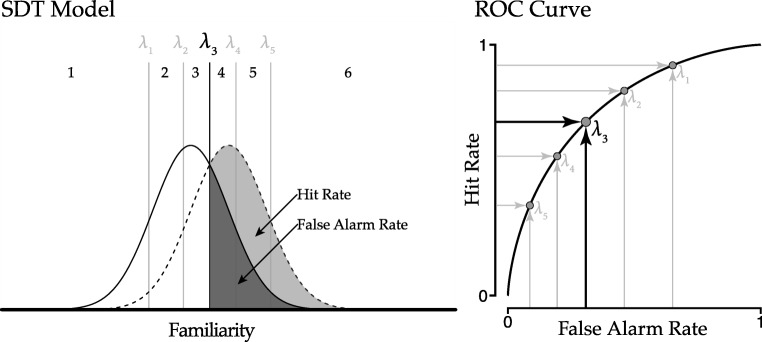
The thresholds parameters, *λ*_*c*_, from the SDT model can be transformed to coordinates of the ROC curve. The hit rate and false alarm rate corresponding to each threshold can be used as coordinates for the ROC curve

A widely used metric to interpret parameter values of the SDT model is the receiver operating characteristic (ROC) curve (Hanley & McNeil, 1982). The ROC curve displays how the hit rate and false alarm rate are affected by changes in thresholds. The translation from the SDT model parameters to the ROC curve is visualized in Fig. 4. Each threshold in the SDT model is associated with a specific hit rate and false alarm rate. For *λ*_3_ the hit rate is the part of the signal distribution shaded light gray, and the false alarm rate is the part of the noise distribution shaded dark gray. This associated mapping can be established for each threshold, resulting in a number of coordinates for the ROC curve. Subsequently, drawing a line through the points leads to the ROC curve.

**Fig. 5:**
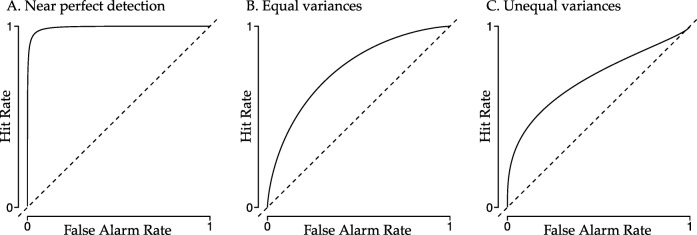
Example ROC curves. The *solid line* represents a theoretical ROC curve. The *dashed line* represents chance performance

Figure 5 shows three example ROC curves. In these graphs, the *x*-axis represents the false alarm rate and the *y*-axis represents the hit rate. Setting the threshold to its lowest possible value will always result in a hit or a false alarm and setting the threshold to its highest possible value will never result in a hit or a false-alarm. Therefore, the ROC curve will always go through [0,0] and [1,1]. The dashed diagonal represents the hypothetical ROC curve if the signal distribution equals the noise distribution, that is, the participant is performing at chance. If the ROC curve is above the dashed diagonal this, means that the participant is performing above chance, and the average strength of the signal exceeds zero.

Panel a in Fig. 5 shows the ROC curve with near perfect detection: the hit rate reaches 1 for low values of the false alarm rate. Panel b shows a typical ROC curve when the signal and noise distribution have equal variances: the curve is symmetrical around the minor diagonal. Panel c shows an ROC curve when the distributions do not have equal variances: the curve is not symmetrical around the minor diagonal.

The mathematical relation between the SDT and ROC parameters is shown in Eq. 7 (Marden, 1996).

7 $$ Z_{\text{HR}} = \frac{Z_{\text{FAR}}}{\sigma^{(s)}} + \frac{\mu^{(s)}}{\sigma^{(s)}}.  $$

Using this equation, the z-transformed hit rate can be calculated using the z-transformed false alarm rate, and the mean and variance of the signal distribution.^3^

## Detection measures

**Fig. 6:**
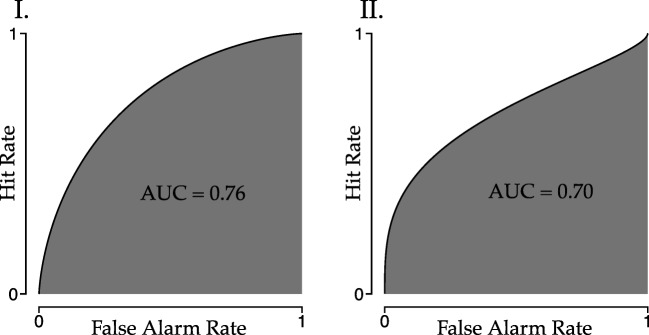
Visualization of area under the curve (AUC) of an ROC curve for the two hypothetical observers. The difference in the variance of the signal distribution is expressed in the difference in the AUC

As we saw in the previous section, the ROC curve is able to accommodate inequality of variances. The ROC curve can easily be converted to a detection measure by calculating the area under the curve (AUC, Wickens, 2001); the larger the AUC, the higher the ability to detect the signal. It is clear that the AUC takes into account the inequality of variances. Also, the AUC will always be between 0.5—if detection is based purely on chance—and 1—if detection is perfect. This makes it straightforward to compare two measurements of the AUC (Fig. 6).

The AUC of the ROC has the attractive property of taking into account differences in variance of the signal distribution between observers, and hence we focus on this measure. The AUC is calculated using Eq. 8 (Wickens, 2001, p. 68), where the noise distribution is assumed to be a standard normal and Φ is the cumulative normal distribution:

8 $$ \text{AUC} = {\Phi}\left( \frac{\mu^{(s)}}{\sqrt{1 + \sigma^{(s)2}}}\right).  $$

## Thresholds

The most important way in which our threshold model improves upon existing confidence ratings SDT models is by estimating the thresholds in a more parsimonious way. Instead of estimating the thresholds individually, which requires one parameter per threshold, the thresholds are modeled using a linear equation. This allows for better estimates of the thresholds in the face of limited data. A consequence of this method is that the threshold placement in our model is restricted to a be linear instead of freely estimated. However, the thresholds can still be placed in a wide variety of ways. Because the threshold model takes into account that observers can set their thresholds in different ways, similar abilities in signal detection can lead to different data, underscoring the difficulties of drawing conclusions directly from the data. To illustrate this point, we performed a simulation study.

**Fig. 7:**
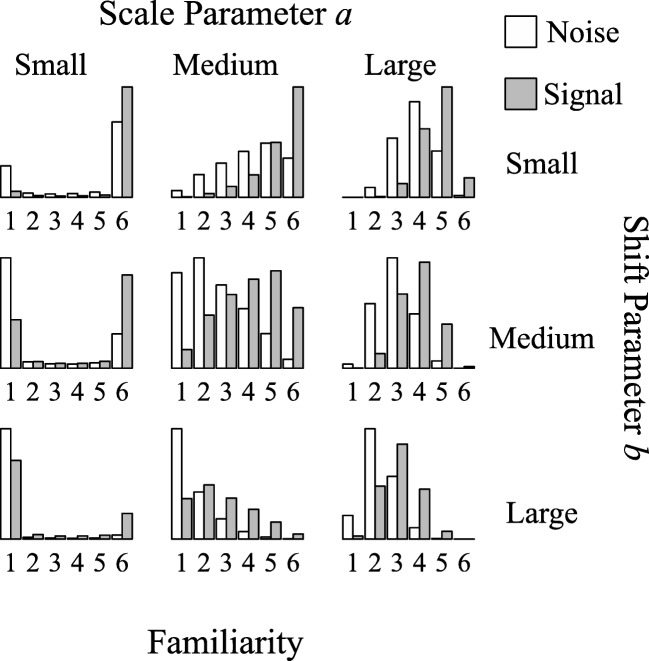
Effect of threshold parameters on familiarity judgments. Nine large datasets (*N* = 10,000) were simulated to visualize the range of model-implied probability distributions over familiarity judgments. The datasets were simulated with the same *μ*^(*s*)^ and *σ*^(*s*)^, but with either a small, medium, or large-scale parameter *a* and either a small, medium, or large shift parameter *b*

To obtain plausible values for the simulation study, we first fitted the threshold SDT model to data from Pratte and Rouder (2011), who gathered confidence ratings on a memory recognition task for 97 participants (this data set is described in more detail below). Based on the estimated parameter values, we chose three values of the scale parameters based on the 1^*st*^, 50^*th*^, and 99^*th*^ percentiles of the estimated values (i.e., *a*_1_ = 0.12, *a*_50_ = 0.84, *a*_99_ = 1.74), and three values of the shift parameters based on the 1^*st*^, 50^*th*^, and 99^*th*^ percentiles of the estimated values (i.e., *b*_1_ = − 0.98, *b*_50_ = 0.14, *b*_99_ = 1.10). We used fixed values of *μ*^(*s*)^ = 1 and *σ*^(*s*)^ = 1 and all possible combinations of the scale and shift parameters to simulate data from the threshold SDT model, resulting in nine different data sets. Figure 7 shows histograms of the simulated data. It is clear that the model can describe various datasets by varying the threshold placement, even when the underlying familiarity distributions are identical.

Figure 7 illustrates that as the scale parameter increases (i.e., moving along the columns from left to right), more answers on the inside of the scale are given and as the shift parameter increases (i.e., moving along the rows from top to bottom), the left side of the scale is used more often. This coverage of possible outcomes makes the model nearly as flexible as having an independent parameter for each threshold while minimizing the number of parameters to estimate.

## Hierarchical extension

The threshold SDT model can be used to fit data from a single observer. However, often there is interest in the detection ability of a group of observers, which requires some sort of aggregation or pooling. One way of pooling is by aggregating the data and then fitting the model on the aggregated data. Another way of pooling is by estimating the parameters for each observer individually and then take the mean or median from these parameter values. Although these methods are computationally simple, they lack a formal model that describes how the group level distribution relates to individual parameter values.

In contrast, in the Bayesian hierarchical approach, individual subject parameters are drawn from a group distribution (Gelman & Hill, 2006). Because the subjects are modeled as part of a group, the individual parameters shrink towards the group mean (Efron & Morris, 1977). The benefit of shrinkage is that the model is much more resistant to overfitting, as the group-level information makes the individual estimates less susceptible to noise fluctuations (Shiffrin et al., 2008). In the hierarchical threshold model, we introduce group distributions for the mean and variance of the signal distribution, and for the scale and shift parameters of the thresholds. The priors for unbounded parameters (mean and shift) are normal distributions whereas the priors for bounded parameters (variance and scale) are either gamma distributions or truncated normal distributions. Exact model specifications and priors are shown in the Appendix^4^.

To confirm the performance of the model we conducted a parameter recovery study. The formal model definitions including prior distributions can be found in the Appendix. First, we fitted the hierarchical SDT threshold model to the data of Pratte et al., (2010) (see next section for a more elaborate explanation). We used the means of the posterior distributions for the individual level parameters *μ*^(*s*)^, *σ*^(*s*)^, *a*, and *b* to generate plausible data. Next, we fit the model to the synthetic data and drew posterior samples from the hierarchical SDT threshold model. Subsequently, we compared the data-generating parameter values to the means of the posterior distributions for the parameter estimates. The correlation between the data-generating parameter values and the recovered parameter estimates was high ($r_{\mu ^{(s)}} = 0.96$, $r_{\sigma ^{(s)}} = 0.90$, *r*_*a*_ = 0.99, *r*_*b*_ = 0.99, see Fig. 15) showing that the hierarchical SDT threshold model has good parameter recovery. More details on this parameter recovery study can be found in the Supplemental Materials at https://osf.io/ypcqn/. The next section applies the model to experimental data.

## Application to experimental data

We fitted the hierarchical SDT threshold model to data from Pratte et al., (2010) who had gathered confidence ratings on a memory recognition task from 97 participants. Each participant studied 240 words—each word for 1850 ms with 250-ms blank periods between two words—randomly selected from a set of 480 words. After the study phase, participants had to indicate how confident they were that a word was part of the study list on a six-point Likert scale (using the ratings “sure new”, “believe new”, “guess new”, “guess studied”, “believe studied”, and “sure studied”) for the whole batch of 480 words. In this experiment, the words in the study list represent the signal items, while the words that were not in the study list represent the noise items.

**Fig. 8:**
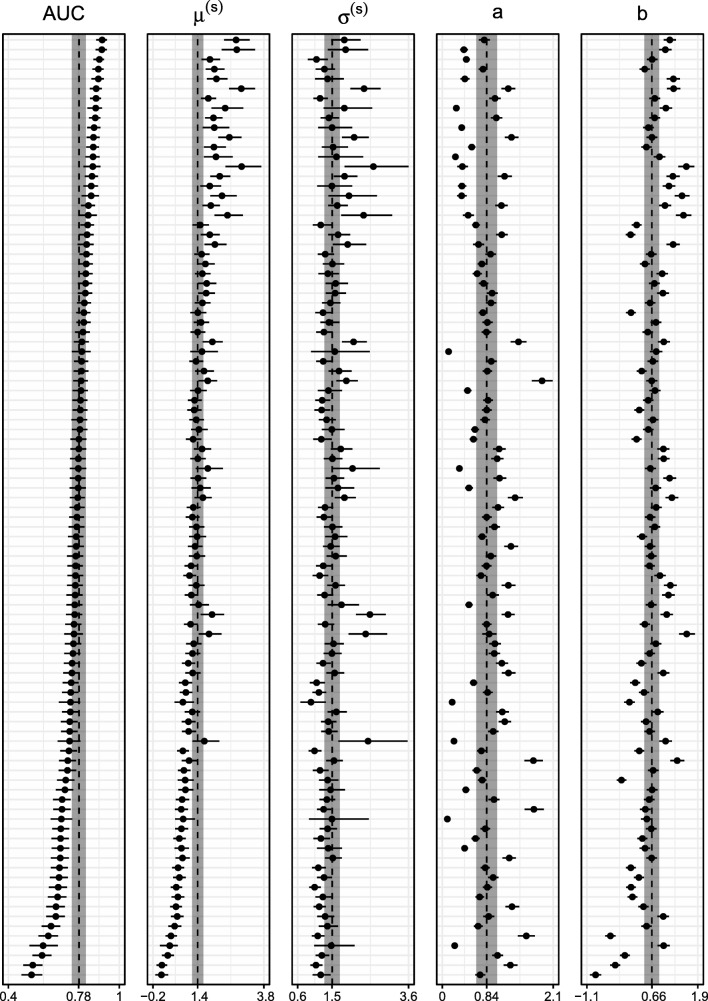
Parameter estimates for all 97 participants from Pratte et al., (2010); the *dot* represents the median and the line represents the 95% central credible interval. The *dashed line* represents the median of the group distribution and the accompanying 95% credible interval is indicated in *grey*

Figure 8 shows the estimated median and 95% credible intervals for each parameter in the model. The dashed vertical line represents the median of the group level estimation with the 95% credible interval shaded gray. The parameters are estimated with a good precision; in general, the credible intervals are narrow. We investigated the fit of the threshold model to the data from Pratte using posterior predictive checks. Although there is some misfit for lower proportions, the model appears to describe the data adequately, see Fig. 16.

**Fig. 9:**
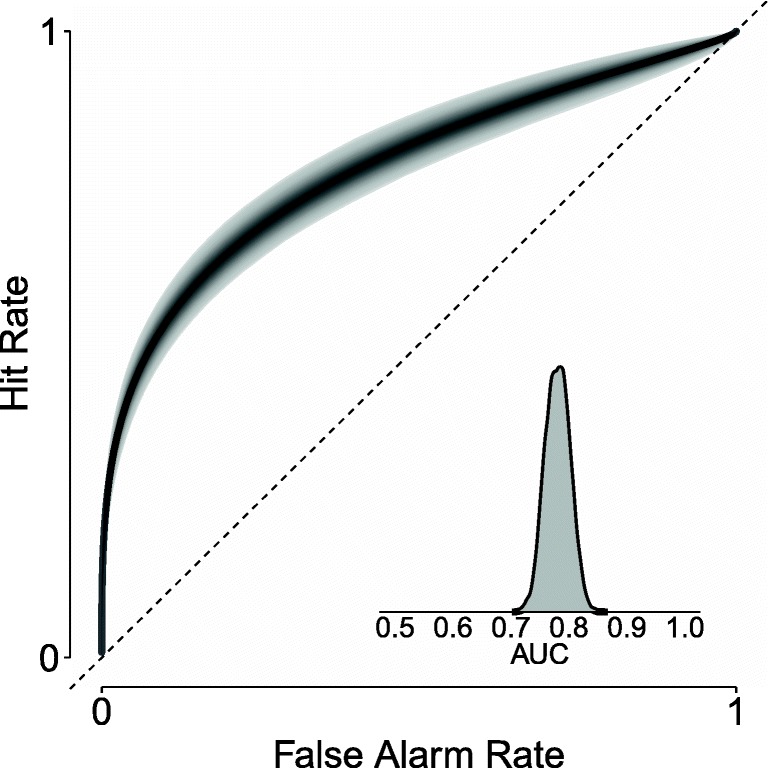
Group level ROC curve with the 95% credible interval in *grey* and the area under the curve (AUC) with the uncertainty in the estimate expressed through the posterior distribution

**Fig. 15:**
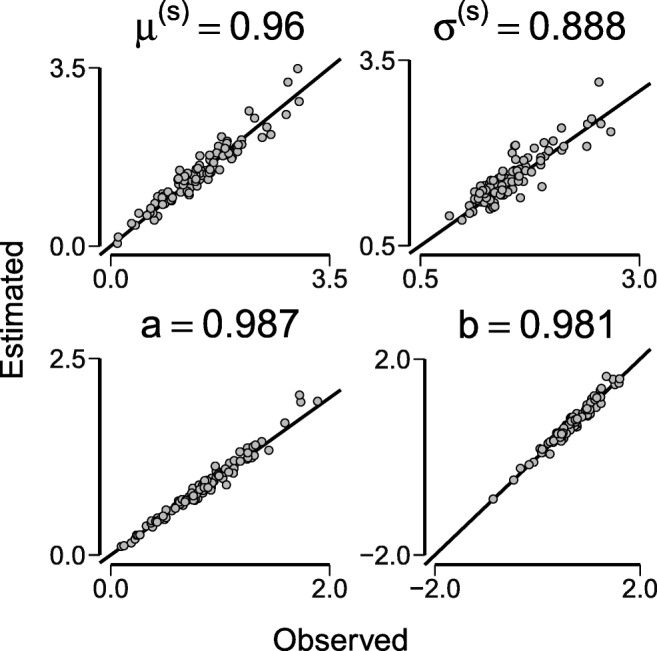
Parameter retrieval of the group level parameters of the simulation study with the hierarchical model

**Fig. 16:**
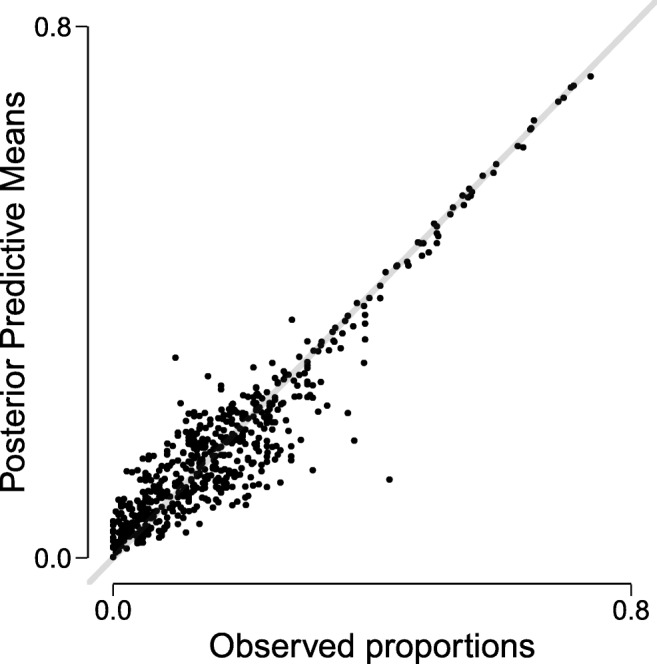
Posterior predictive check for the data from Pratte et al., (2010). Observed proportions of a rating per person (*x*-axis) versus posterior predictive means of the model (*y*-axis). The model fits ratings with a higher observed proportion better than those with a lower observed proportion. This occurs because those ratings constitute more observations and are weighed more by the likelihood. Lower proportions are captured less well by the model. Likewise, the lower proportions are based on less data and are therefore more noisy

The model parameters can also be used to produce an ROC curve. Figure 9 shows the ROC curve for the group level, where the shaded area represents the uncertainty in the estimate, and the density plot shows the posterior distribution for the AUC. Note that the uncertainty in the ROC and the AUC is induced by the uncertainty in the model parameters.

## Discussion

The threshold SDT model describes how people estimate the familiarity of signal and noise items. The main contribution of the model is that it provides a parsimonious way of estimating the thresholds instead of sacrificing one parameter per threshold. We also showed how this model can be applied to experimental data. This paper presents a first effort in parsimonious threshold estimation that should be applicable to many SDT applications. It can also be used as a starting point for more complicated applications of SDT models. A straightforward empirical test of the threshold SDT model is to examine how experimental manipulations map onto the model parameters. For example, one may conduct a test of specific influence and examine the extent to which effects of changes in base-rate are absorbed by the threshold *a* and *b* parameters.

Because the threshold SDT model features only four parameters, it is relatively straightforward to add other effects, e.g., the item effects mentioned in the discussion of Pratte and Rouder (2011). For example, a researcher could hypothesize that there is a difference in response bias between two conditions, and that this difference maps onto the shift parameter. To incorporate this into the model, Eq. 5 could be modified to include a covariate on the shift of the thresholds. Such a modification is identical to adding a predictor to a regression model. This allows for relatively easy group comparisons; in contrast, such comparisons are difficult for models that require one parameter per threshold, as multiple estimates need to be considered simultaneously.

Expanding the transformation of the thresholds into a linear model introduces the need for model comparison. To assess the relevance of a predictor, one compares a model without the predictor to a model with the predictor. Within the Bayesian framework, comparing models is often done by means of Bayes factors (Mulder & Wagenmakers, 2016; Jeffreys, 1961). Although no analytical formulas exist for calculating Bayes factor for SDT models, an approximation can be obtained using numerical techniques on the obtained MCMC samples, e.g., via bridge sampling Gronau et al., (2017) and Meng and Wong (1996).

In sum, the threshold SDT model provides a parsimonious and straightforward account of confidence rating data, allowing researchers to quantify not only discriminability but also confidence category thresholds. The uncertainty in the model’s parameter estimates can be used to induce uncertainty in crucial SDT measures such as the area under the ROC curve.

## Figures and Tables

**Fig. 10:**
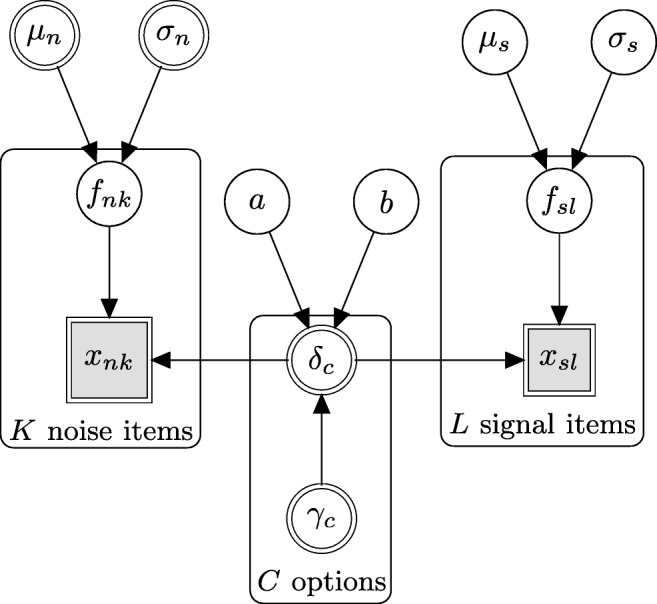
Graphical model representation of the SDT threshold model

**Fig. 11:**
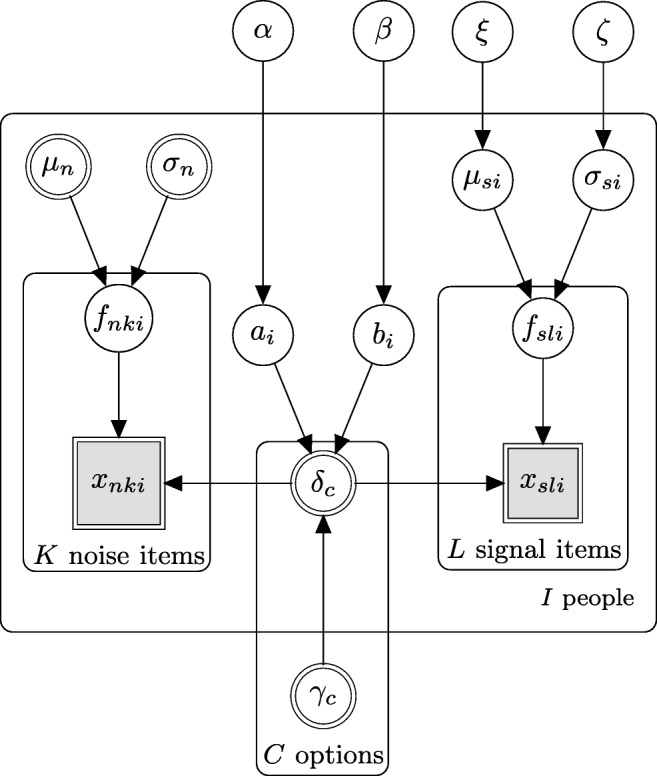
Graphical model representation of the hierarchical SDT threshold model

**Fig. 12:**
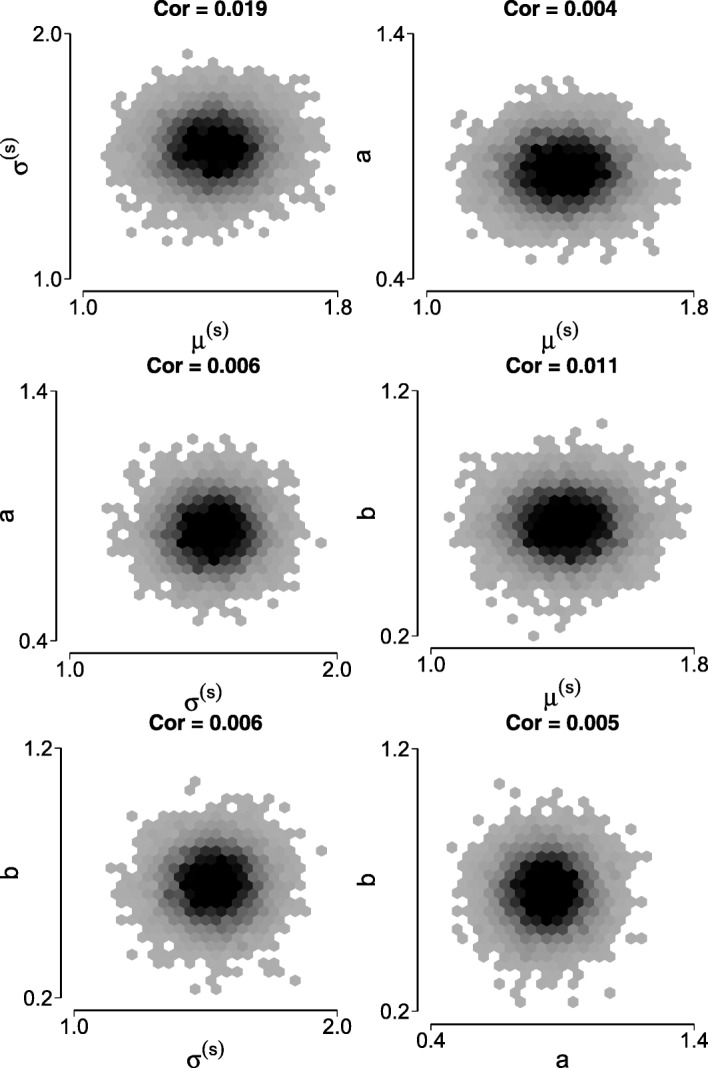
Bivariate hex plots of the group-level parameters. A *brighter color* indicates a higher frequency of samples. The Pearson correlation between the posterior samples is shown on top of each panel. Note the negligible trade-off between the parameters

**Fig. 13:**
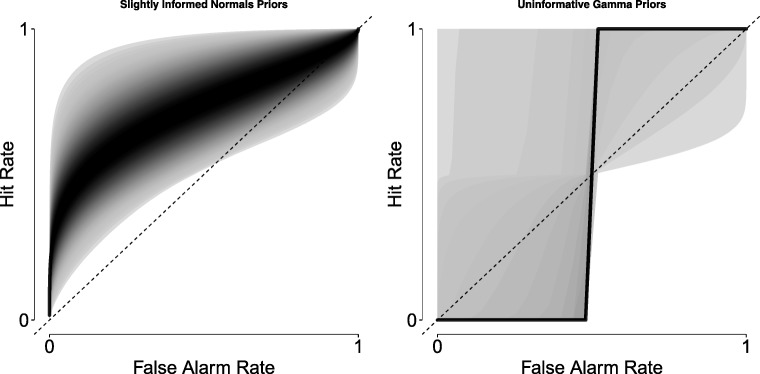
Prior predictive ROCs for the proposed priors (*left panel*; see Fig. 11 for the priors) versus the standard uninformative gamma priors (*right panel*; $\alpha , \beta , \xi , \zeta \sim \text {Gamma}(0.01, 0.01)$)

**Fig. 14:**
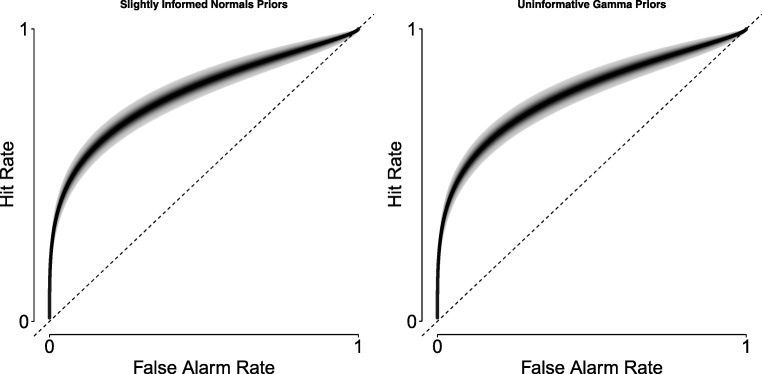
Posterior predictive ROCs for the proposed priors (*left panel*; see Fig. 11 for the priors) versus the standard uninformative gamma priors (*right panel*; $\alpha , \beta , \xi , \zeta \sim \text {Gamma}(0.001, 0.001)$)

## Appendix

This Appendix contains both the formal BUGS model definition and the graphical representation of the SDT threshold model and the hierarchical SDT threshold model. The R code that calls the BUGS code is available at https://osf.io/v3b76/. The model definition and graphical representation define all priors and relations between parameters and data. For more information on the BUGS modeling language and the graphical representation of these models, see Lee and Wagenmakers (2013).

## Footnotes

1 For the translation we used a logistic quantile function. Other choices, such as a Gaussian quantile function, are also possible.

2 The individual values for the parameters were drawn from: *μ*_*s**i*_, normal distribution with mean 1 and standard deviation 0.5 truncated at [0, 3], *σ*_*s**i*_, normal distribution with mean 1 and standard deviation 0.5 truncated at [1, 3], *a*, gamma distribution with shape parameter 2 and rate parameter 2, and *b*, normal distribution with mean 0 and standard deviation 0.5.

3 Note that z-transformed ROC functions are linear. In addition, when the equal variance assumption is met, the slope is one. When the variance of the signal distribution is larger than that of the noise distribution, as is generally found to be the case in recognition memory, the slope is less than one.

4 We opted not to use highly uninformative priors as the resulting prior ROC curves are implausible. See Figs. 13 and 14 for the prior and posterior ROC curves under slightly informed and highly uninformative priors. Different priors had negligible effect on the posterior distribution, see the Supplemental Materials Figures on https://osf.io/ypcqn/ for a comparison.
